# Revolutionizing Three-Dimensional Printing: Enhancing Quality Assurance and Point-of-Care Integration through Instrumentation

**DOI:** 10.3390/pharmaceutics16030408

**Published:** 2024-03-16

**Authors:** Javier Suárez-González, Eduardo Díaz-Torres, Cecilia N. Monzón-Rodríguez, Ana Santoveña-Estévez, José B. Fariña

**Affiliations:** 1 Departamento de Ingeniería Química y Tecnología Farmacéutica, Campus de Anchieta, Universidad de La Laguna (ULL), 38200 La Laguna, Spain; jsuarezg@ull.edu.es (J.S.-G.); cmonzonr@ull.edu.es (C.N.M.-R.);; 2 Instituto Universitario de Enfermedades Tropicales y Salud Pública de Canarias, Universidad de La Laguna (ULL), Avenida Astrofísico Francisco Sánchez, s/n., 38200 La Laguna, Spain; 3 Programa de Doctorado en Ciencias Médicas y Farmacéuticas, Desarrollo y Calidad de Vida, Universidad de La Laguna, 38200 La Laguna, Spain

**Keywords:** 3D printing, additive manufacturing, semi-solid extrusion, extrusion dynamics, printing speed, temperature control, quality assurance, point-of-care applications, formulation study, printlet

## Abstract

Three-dimensional printing in the field of additive manufacturing shows potential for customized medicines and solving gaps in paediatric formulations. Despite successful clinical trials, 3D printing use in pharmaceutical point-of-care is limited by regulatory loopholes and a lack of Pharmacopoeia guidelines to ensure quality. Semi-solid extrusion is a 3D printing technology that stands out for its versatility, but understanding the fluid dynamics of the semi-solid mass is critical. The aim of this research is to look into the advantages of instrumenting a 3D printer with a semi-solid extrusion motor-driven printhead, which is able to record the printing pressure over time, for in situ characterization of the semi-solid mass and quality evaluation of dosage forms. Four formulations using hydrochlorothiazide as the active pharmaceutical ingredient and several excipients were used. Their flow properties were studied at different printing speeds and temperatures using traditional techniques (rheometer and Texture Analyzer) and the proposed semi-solid extrusion motor-driven printhead incorporated into a printing platform. In addition, the influence of printing speed in the printing process was also evaluated by the study of printing pressure and printlet quality. The results demonstrated the similarities between the use of a Texture Analyzer and the semi-solid extrusion motor-driven. However, the latter enables temperature selection and printing speed in accordance with the printing process which are critical printing parameters. In addition, due to the incorporation of a sensor, it was possible to conclude, for the first time, that there is a link between changes in essential printing parameters like printing speed or formulations and variations in printing pressure and printlet quality attributes such as the energy require to obtain a single dosage unit, weight or diameter. This breakthrough holds a lot of potential for assuring the quality of 3D printing dosage forms and paving the way for their future incorporation into point-of-care settings.

## 1. Introduction

Additive manufacturing, or three-dimensional printing (3DP), is a rapidly evolving technology that offers flexibility and diversity to the field of individualised medicine. It is possible to provide quality dosage forms (printlets) when a child-friendly medicine is not commercially available for treating paediatrics [1,2,3]. Because of its wide applicability, healthcare professionals are aware of the benefits and prospects of 3DP for the production of individualised medicines and their incorporation in point-of-care settings [4,5]. In this sense, clinical studies have already carried out on the elaboration in a hospital and sequential administration of chewable printlets for treating Maple Syrup Urine Disease in paediatric patients [6,7]. However, the incorporation of 3DP in point-of-care setting has not advanced, mainly due to the absence of a regulatory framework. There have been tentative steps to address this situation, but mainly related to 3DP of medical devices and not the elaboration of dosage forms for pharmaceutical applications [4,8,9,10]. In addition, United States Pharmacopoeia (USP) or European Pharmacopoeia has not provided recommendations regarding the quality control of dosage forms produced by 3DP. Hence, traditional Pharmacopoeia tests for finished products have been carried out for their quality evaluation [7,11,12].

A relevant number of 3DP technologies, including extrusion-based (direct powder extrusion (DPE), melt extrusion deposition (MED), fused deposition modelling (FDM), and semi-solid extrusion (SSE)), powder-based (binder jetting and powder bed fusion), liquid-based (vat photo-polymerization and material jetting) and sheet lamination-based systems, have been tested in the pharmaceutical industry [13,14]. From all previously mentioned technologies SSE is able to develop printlets based on the extrusion of a semi-solid mass (gel or paste) contained in a syringe through the application of air-pressure or using a screw gear rotation. This leads to the deposition, layer by layer, of the extruded mass on the printing platform. Such technology has relevant advantages such as the possibility of producing printlets with a high drug load, the potential use of a broad range of excipients, and the option of using lower temperatures, which make it perfect for thermolabile active pharmaceutical ingredients (API), as well as portability and a competitive price in comparison with the rest of techniques [13,15]. Furthermore, this technology makes it simple and easy to decentralise the preparation of ready-to-use printing cartridges (combination of semi-solid mass and syringe) with different API and excipients, being a viable option in point-of-care settings. In addition, small steps have already been taken in this direction enabling the elaboration of efavirenz printlets using pre-elaborated inks and DPE [7].

Despite all the advantages previously mentioned, one critical quality attribute (CQA) of the semi-solid mass is the flow properties and their influence on the extrudability profile, binding between layers, and ability of the material to hold its shape and withstand the upper layers [16]. All these items can be enclosed in the study of the printability of the printing cartridge. These materials have already been extensively studied using an oscillating rheometer and texture analyser (TA), but it was determined that data from the latter were more accurate in forecasting relevant variables such as printing pressures, as there is a resemblance to SSE printing conditions [17]. As a result, the extrudability profiles will provide critical information such as extrusion pressure, yield stress, steady flow, and so on, which will define the extrudability and flowability of the semi-solid mass and, ultimately, the printlet quality.

In SSE, 3DP pressure applied to the top of the syringe is gradually increased until material begins to flow through the nozzle and keeps increasing until the necessary flow rate is reached, at which point it must remain constant without excessive oscillations to ensure the dose of API and the quality of the finished product. In the case where a printing control is established, situations where the nozzle is clogged can be detected as an increase in applied pressure. In addition, the presence of air inside the syringe or nozzle will be detected as a decrease in pressure, which will produce poor-quality printlets in terms of weight and, consequently, API dose [16,18,19,20,21,22].

Such is the importance of monitoring and controlling printing pressure that the research group created and patented an innovative pressure instrumentalized SSE motor-driven printhead (SSE-P) which is able to record the printing pressure over time [23]. This component is the first step to introduce a process analytical technology (PAT) strategy, which enables in-line manufacturing process control and detects dose units with poor-quality features in SSE 3DP [24]. However, this SSE-P is not only capable of monitoring printing pressure, but it can be also used to study the extrudability profile of semi-solid masses, as with a traditional TA which, as previously mentioned, resembles the printing process. In addition, this SSE-P, as part of the printing platform, would be capable of introducing printing conditions during the evaluation of the extrudability profile, such as changes in temperature, speed or shrinkages, that could be relevant during printing process.

The objective of this work is to analyse the implications of the instrumentalization of a 3D printer with an SSE-P sensor for the in situ study of the extrusion dynamics of semi-solid masses and the detection of possible critical printing parameters through the evaluation of their printability in combination with in-line and off-line quality controls.

## 2. Materials and Methods

### 2.1. Materials

Hydrochlorothiazide (HCTZ), polyvinylpyrrolidone K30 (PVP), and oral banana essence were purchased from Acofarma (Barcelona, Cataluña, Spain). Banana flavouring essence was used to mask the slightly bitter taste of HCTZ to improve drug adherence in the paediatric population [25]. Ac-Di-Sol (Croscarmellose Sodium) was acquired from FMC Corporation (Philadelphia, PA, USA). Lactose monohydrate was obtained from Sigma-Aldrich (St. Louis, MO, USA). Purified water was obtained from a water purification system (Puranity TU 12, VWR, Radnor, PA, USA).

### 2.2. Preparation of the API-Loaded Feedstock Formulations

A total of four semi-solid formulations were prepared containing HCTZ as model drug and varying the proportion of excipients as shown in Table 1. For the preparation of each of the semi-solid masses all the components were milled separately using a mortar and pestle. Each component was then added to a Unguator^®^ (Microcaya, Bilbao, País Vasco, España, Spain) mixer jar in the proportions listed in Table 1 and in the following order: HCTZ, lactose monohydrate, PVP, banana flavouring essence and croscarmellose sodium. Then, 2 mL portions of water were subsequently added, followed by mixing periods of 15–20 s at 500–700 rpm, until the proportion of purified water was achieved.

### 2.3. Flow Properties of the Formulations

Flow properties of the formulations were studied using well-known systems such as an Oscillatory rheometer and a TA, the results of which were compared with the instrumentalized 3D printer with SSE-P sensor.

#### 2.3.1. Oscillatory Rheometer

Flow properties of each formulation were studied in triplicate using a rheometer Bohlin CS (Bohlin Instrument Inc., Cranbury, NJ, USA) equipped with a Peltier and steel parallel plate of 20 mm diameter. A total of three tests were performed for each of the masses, using the Oscillation mode of the equipment (frequency and amplitude sweep) and using the Creep recovery mode. For all the tests, the gap and the temperature were set at 0.6 mm and 40 °C, respectively, mimicking printing conditions.

Oscillation frequency sweep tests were carried out at a shear stress of 50 Pa, with an oscillatory frequency of 0.01 Hz to 100 Hz. Oscillation amplitude sweeps tests were performed at a frequency of 1 Hz, with an oscillatory shear stress range of 0.2387 Pa to 10,000 Pa. For measuring the recoverable characteristics of the semi-solid formulations, a creep recovery test was performed for a total of three cycles in which a shear stress of 500 Pa was applied for 60 s, the approximate time it takes to obtain a printlet of 10 mg, followed by a subsequent time of 60 s without applying shear stress, where the recovery of the analysed material was evaluated. Data collection was performed using the Bohlin software package (version R6.51.0.3, Malvern Instruments, Worcestershire, UK) of the instrument.

#### 2.3.2. TA

**Figure 1:**
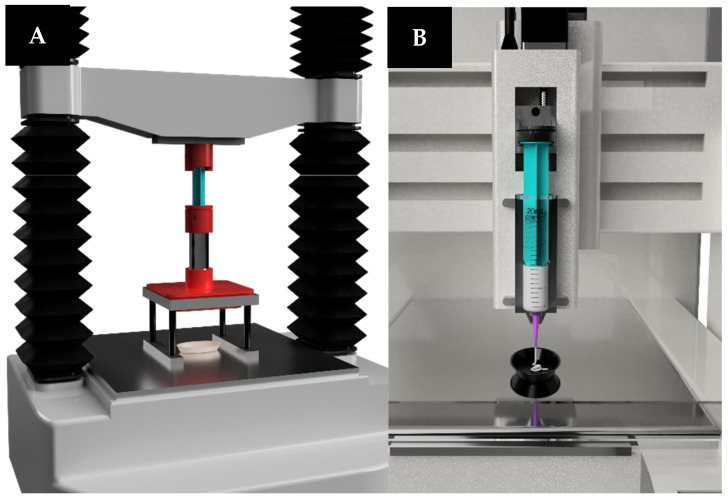
Equipment adapted for use in the characterisation of semi-solid mass. (**A**) Texture analyser; (**B**) instrumentalized semi-solid extrusion motor-driven printhead.

An extrudability profile was performed using a TA adapted for the proposed task (TA.HDplusC, Stable Micro Systems Ltd., Surrey, UK) in a compression mode obtaining the applied force as a function of time, Figure 1A. For this purpose, 10 g of each semi-solid formulation was filled into 20 mL Luer-Lock syringe (B. Braun Medical Inc. OEM, Melsungen, Germany), commonly used by SSE-P of the pharmaceutical 3DP platform (M3DIMAKER, FabRx Ltd., London, UK) [23]. To perform the different extrusion cycles, the syringe was equipped with a tapered plastic nozzle with an inner diameter of 0.61 mm (20 Gauge) (Fisnar QuantX™, Germantown, WI, USA). In order to study how the printing speed affects the physical properties of a semi-solid formulation, a compression/shrinkage cycle was set up (see Table 2).

Plunger displacement speed (PDS) was estimated using Equation (1):

(1) $$\mathrm{PDS}=\frac{{\mathrm{LH}}^{2}}{D^{2}}\cdot \mathrm{TPS}$$

where TPS is the target print speed, LH is layer height, and D is syringe inner diameter.

All measurements were performed at 25 °C for each semi-solid formulation. Data collection and calculation were carried out using the Texture Exponent 32 software package (version 5.0.8.0, Stable Micro Systems Ltd., Surrey, UK) of the instrument. Every measurement on each semi-solid formulation of the fractional factorial design was performed in triplicate. With an empty syringe, compression/shrinkage cycles were carried out for each parameter combination in order to capture a baseline that may be used to rectify the signal that is only obtained from the friction of the polypropylene plunger against the syringe walls.

As the applied force in Newtons as a function of time is obtained for each formulation and printing speed, pressure values were obtained taking into account the area of the tip. Then, yield point, as the pressure where the semi-solid mass starts to flow, was obtained for each run as well as relevant variables such as Young Modulus, shear stress in the steady flow, shear rate, apparent and dynamic viscosity. Young Modulus shows the capacity of certain materials to resist elastic deformation and was calculated as the slope from the pressure–distance plot before yield point [2].

Shear rate was calculated following Equation (2) [26,27]:

(2) $$\gamma =\frac{4Q}{\pi r^{3}}$$

where *r* is the nozzle radius (mm), and *Q* is the volumetric flow rate (mm/s) calculated as the printing speed multiplied by the radius of the syringe (mm).

In addition, shear stress in the steady flow was calculated using data from force in the steady flow, taking into account the area of the tip. Apparent viscosity was obtained from the quotient between shear stress and shear rate, and dynamic viscosity was estimated from Equation (3) [28]:

(3) $$F=\frac{128Q\mu LA}{\pi D^{4}}$$

where *Q* is the volumetric flow rate, that in the steady flow was considered as the printing speed, *μ* is the dynamic viscosity, *A* is the syringe area, and *L* and *D* are the length and diameter of the tip.

#### 2.3.3. SSE-P

A 3DP pharmaceutical platform (M3DIMAKER, FabRx Ltd., London, UK) equipped with an SSE-P was used to characterise the physical properties of the semi-solid formulation described above, Figure 1B. To achieve this feature, the original syringe plunger used by the printing platform was replaced by an instrumented pressure-measuring plunger developed by the research group. This sensor was calibrated following the guideline DKD-R 6-1 for the calibration of pressure gauges published by the German Calibration Service under the patronage of the Physikalisch-Technische Bundestalt using certified reference standards [29].

Analogously to what was performed in the TA, different tests were set up in which different printing conditions were simulated by varying the printing temperature (25 °C and 40 °C) and printing speed (10 mm/s, 20 mm/s, 30 mm/s). Using a data capture system installed in the printer, other variables such as the displacement of the plunger holding system or the weight of extruded mass using a precision scale (ENTIRIS153I-1S, d = 0.001 g, Sartorius, Goettingen, Germany) as a function of time were recorded. As mentioned in the previous section, a signal derived exclusively from the friction of the instrumented plunger against the syringe walls was quantified, and the same variables were studied.

### 2.4. Printability and Fidelity Test

#### 2.4.1. Three-Dimensional Design and Slic3r Profiles (Printing Settings)

A 3D model file (.stl file) was generated after a pattern of 60 printlets, in a cylinder form with a 6.00 mm radius and a 2.40 mm height, each spaced by 1 cm, was designed using Autodesk^®^ Fusion 360 (version 2.0.9011, Autodesk Inc., San Francisco, CA, USA). The 3D models were sliced with the open source Repetier Host software (version 2.1.6, Hot-World GmbH & Co. KG, Willich, Germany) with Slic3r (version 1.3.1-dev, GNU Affero General Public License). The gcode file obtained was automatically modified using a python script to randomly change the printing speed, to finally have 20 dosage forms printed with each of the speeds (10 mm/s, 20 mm/s, 30 mm/s). The rest of the printing parameters remained unchanged (layer height: 0.61 mm, Shells: 2, Infill: 70%, Nozzle temperature: 40 °C). In addition, for all speeds 10 printlets were developed at 5 mm/s to ensure previous pressurisation of the syringe and avoid possible material deposition errors.

#### 2.4.2. Three-Dimensional Printing of the Orodispersible Printlet

The 3DP pharmaceutical platform (M3DIMAKER, FabRx Ltd., London, UK) equipped with an SSE-P was used to execute the design gcode file for each of the previously tested semi-solid formulations. A 20 mL Luer-Lock syringe with a tapered plastic nozzle with an inner diameter of 0.61 mm (20 Gauge) were used for the printing process. The original syringe plunger was replaced by the self-designed instrumentalized plunger described above, in order to record as PAT the pressure used during the entire printing process.

#### 2.4.3. Printing Process and Printlet Quality Control

As part of the in-line quality control and PAT strategy printing process was under control due to the SSE-P sensor installed on the printing platform. Once completed, a Python script, designed by the research group, was used to relate each variable controlled, for example, pressure, with a specific dosage unit. Then, for each printlet, a complete report of the printing pressure, the total area under the curve (AUC) from the force-time plot, temperature, humidity of the printing platform, etc., was obtained. This information allows the operators to detect the presence of air or obstructions that could affect the CQA of the dosage units, as it can be plotted in a 3D scatter representation for each coordinate in space (X,Y,Z).

After completing the printing process, printlets were stored under room temperature and protected from light for 6 h to ensure water evaporation as part of the drying process.

Subsequently, and as part of the off-line quality control process, each dosage unit was weighed using a precision scale (XSR105DU, d = 0.01 mg, Mettler-Toledo GmbH, Greifensee, Switzerland), being able to study the variation in mass for each printing speed.

In addition, two pictures, were taken before and after the drying process, of the entire printlet batch produced with each formulation (iPhone 14 Pro, Apple, Cupertino, CA, USA). Both images were then analysed to measure each printlet and detect defects at the edges of the printlet, using a properly developed Python script with the OpenCV computer vision image analysis library (open-source Apache 2 License).

Finally, with the help of a differential scanning calorimeter (DSC25, TA instruments, Newcastle, DE, USA) that had been previously calibrated with indium, samples of API, excipients, wet-mass, and crushed printlets were thermally examined. Next, 5 mg samples were weighed and hermetically sealed in an aluminium pan for examination. Equilibrating at 0 °C, the starting temperature was increased to 300 °C at a rate of 10 °C/min. TRIOS 5.1.1.46572 analysis software (TA instruments, Newcastle, DE, USA) was used to collect the data and plot it as a temperature (°C) vs. normalized heat flow (W/g) curve.

### 2.5. Statistical Analysis

A statistical test was carried out to compare the results from rheometer, TA and SSE-P as well as to evaluate the influence of printing speed in the process and quality of dosage unit. Statistical analyses were carried out by SPSS^®^ for Windows^®^ (22.0; SPSS Inc., Chicago, IL, USA), with 0.05 as significance level.

## 3. Results

### 3.1. Flow Properties of the Formulations

#### 3.1.1. Oscillatory Rheometer

**Figure 2:**
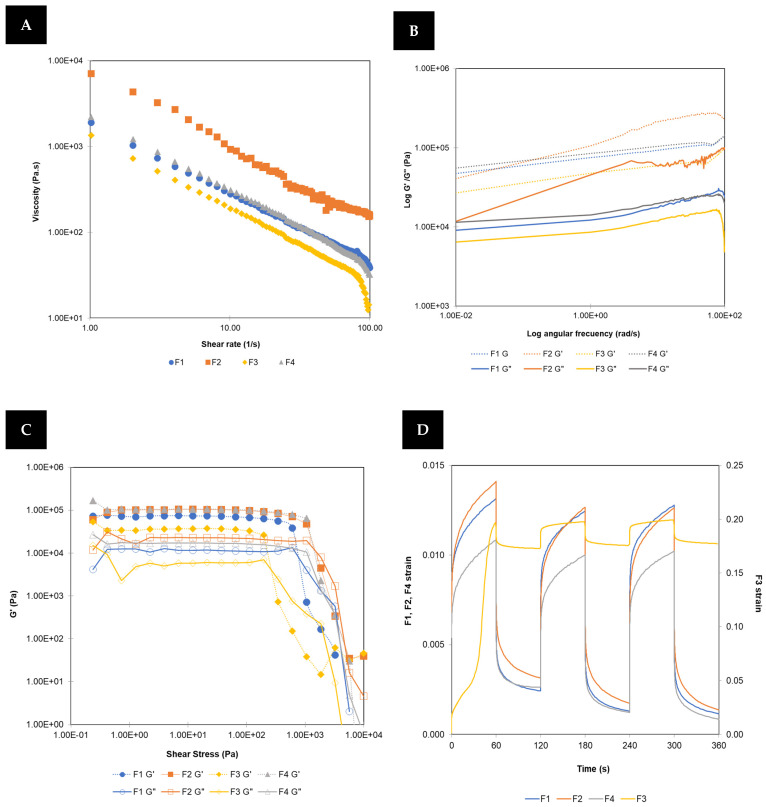
Flow test (**A**), frequency sweep (**B**), amplitude sweep (**C**), and creep recovery (**D**) test for all formulations studied by oscillatory rheometer.

Understanding the rheological characterization is essential for predicting the printability behaviour of masses during 3DP. Figure 2A shows the characteristic flow profile of non-Newtonian fluids, where the viscosity of all formulations decreases as the shear rate increases [30]. The results of the frequency sweep test show G′ and G″ values that are parallel for each mass during the increase in frequency (see Figure 2B), characteristic of a solid gel-like behaviour [31]. This type of behaviour is defined as a non-fluid colloidal network or a polymeric network which expands throughout its volume by a fluid and has a finite and usually low yield stress [32].

The printability and shape fidelity of the printable form were analysed by an amplitude sweep test. The results show that in all cases, there is an almost constant value of G′ greater than G″ (Figure 2C), indicating a solid gel-like behaviour [30]. F3 presents a G′ value slightly lower than that of the rest of the studied inks.

The yield stress was calculated as the point where G′ loses its linearity, and the flow stress as the midpoint of the crossover between G′ and G″ [30]. As can be observed in Table 3, the values of both parameters are the same for F2 and F4 inks, and these values are higher than for F1 and F3 inks. Regardless of the difference in the composition of the masses between disintegrant and diluent, where essence is not included in their composition, interactions formed between components create stronger networks, providing more robust printable structures [33]. F1 ink behaviour is similar to F2 and F4 inks, but the addition of the essence reduces the values of yield stress and flow stress, improving the ink flow but decreasing the robustness of the printable forms. For F3 ink, with lower proportion of disintegrant and equal amount of essence as F1 ink, it does not form a stronger network as F2 and F4 inks, exhibiting greater fluidity, which provides easier printing process but less robust printlets. F3 is the ink that flows more but will provide less robust printlets.

The creep recovery test is based on simulating the printing conditions of every printlet. In this way, shear stress is applied during the printing time (60 s), and then stopped, and the cycle is repeated using the same time interval as the printer. This records the ink displacement that occurs during the elaboration of every printlet during the process, as expected from the results obtained in the rest of the rheological tests (see Figure 2D). As can be seen, F1, F2, and F4 show an adequate thixotropic response for 3DP process. F3 ink is the mass that suffers the greatest initial displacement, and subsequently, in each cycle, recovery is also slightly higher than that of the other inks, whose behaviour in this test is very similar. It is possible that this different F3 behaviour is due to the shear stress value applied (500 Pa), which is above its yield stress value.

#### 3.1.2. TA and SSE-P Comparison

**Figure 3:**
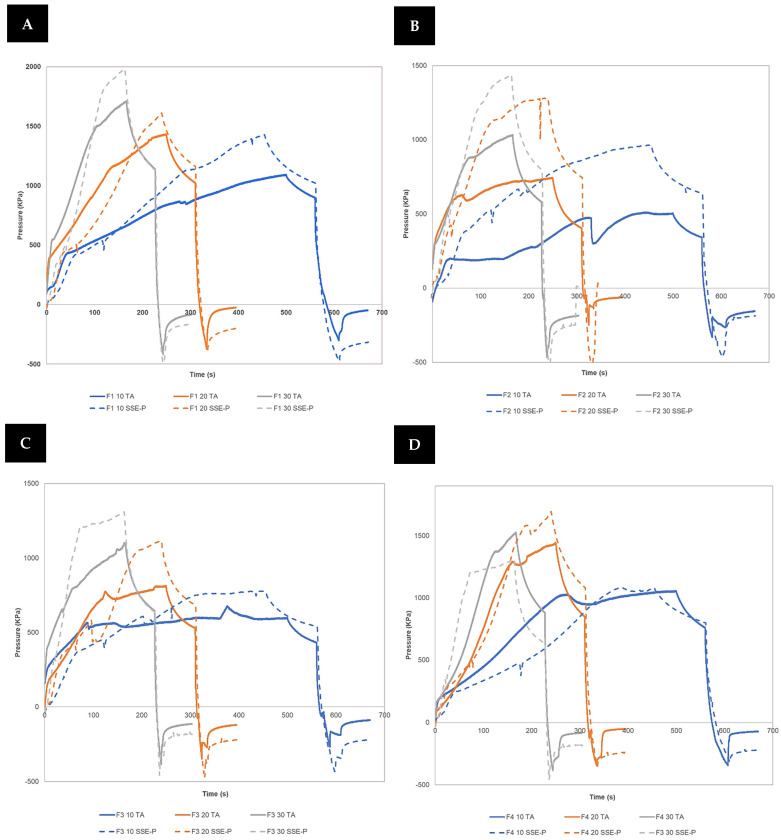
Characterization of F1 (**A**), F2 (**B**), F3 (**C**), and F4 (**D**) at different printing speeds using TA and SSE-P.

In Figure 3, a graphical representation of the characterization of all formulations at 25 °C and different printing speeds using a TA and the 3D printer pressure instrumentalized SSE-P is shown. As may be seen, both tools showed a similar profile for the formulations all, but the signal of SSE-P is slightly higher.

In Table 4 and Table 5, a comparison between parameters studied for TA and the 3D printer pressure instrumentalized SSE-P is shown for every formulation and printing speed.

A linear regression showed that there was a statistically significant relationship for all parameters between the values obtained for TA and SSE-P (*p* < 0.05; r > 0.7). In the case of the Young Modulus, there is no significant correlation between tools due to the uncertain determination of the yield point in the case of the use of TA compared with SSE-P. Therefore, the use of the SSE-P is equivalent to the use of the traditional TA, and it can be used to characterize semi-solid masses prior to being printed.

In addition, this new instrumentalized printhead has the possibility of replicating what happens on the printing platform, changing with precision the sample temperature and evaluating the influence of this parameter in the material’s flow behaviour. This is extremely important in SSE 3DP as a certain temperature might be needed to extrude a semi-solid mass, generally 20–40 °C.

**Figure 4:**
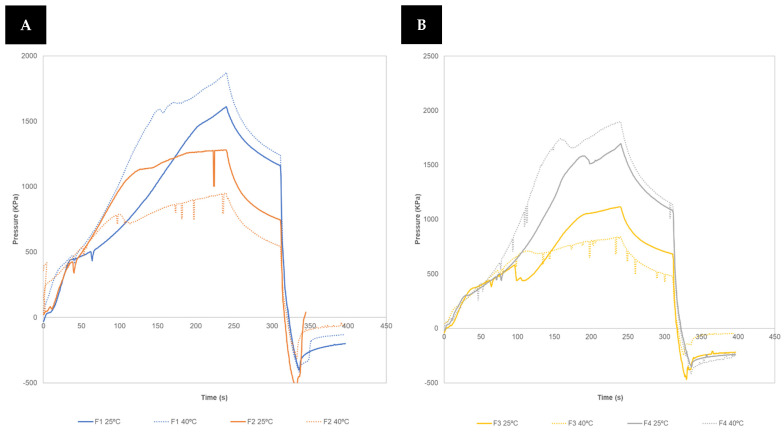
Variation in extrusion profile of the formulations when an increase in temperature is applied. (**A**) F1 and F2 at 25 and 40 °C. (**B**) F3 and F4 at 25 and 40 °C.

Indeed, as can be seen in Figure 4 a significant change in material behaviour at the same printing speed (10 mm/s) was detected for all formulations when a temperature of 40 °C was applied. The signal for those formulations with a higher percentage of croscarmellose sodium (F1 and F4) is higher at 40 °C than at 25 °C, which is contrary to the other formulations (F2 and F3). The latter might be related with the higher solubility of lactose at 40 °C, reducing the flow resistance, in comparison with 25 °C [34]. In the case of F1 and F4, it might be related to the increase in viscosity due to changes in cellulose network [35].

For evaluating the influence of printing speed on several dependent variables of the semi-solid mass which can be studied using SSE-P (yield point, yield point time, maximum pressure, pressure at flow cessation, Young Modulus, shear stress at steady flow and apparent/dynamic viscosity), the results of the Multivariate Analysis of Variance showed a significant statistical relationship (*p* < 0.05) for all tests performed: Wilks’ lambda (0.010), Pillai’s trace (0.027), and Roy’s greatest root (<0.01). Consequently, it is possible to demonstrate that the printing speed affects all the variables that have been researched when they are considered worldwide. Individually, the yield point does not change with the increase in printing speed as the pressure depends solely on their composition, but it does change the time required to reach that pressure; lower pressure at higher printing speeds. In the case of maximum printing pressure, this increases as an indicator of the increment of the SSE-P’s displacement per unit of time and the accumulation of the force transmitted to the semi-solid mass. Apparent and dynamic viscosities decrease as printing speed increases because of the increase in shear rate and volumetric flow rate, respectively.

This analysis was not performed for TA results, as in this case, fewer dependent variables can be obtained; the degrees of freedom are below the necessary level.

In view of the results, temperature and printing speed should be considered as critical 3DP parameters, as relevant variables such as printing pressure or yield point can be modified, and are therefore essential to control and optimise the printing process.

#### 3.1.3. Oscillatory Rheometer and SSE-P Comparison

In Table 3, a comparison between values for yield point obtained from the rheometer and SSE-P are shown at 40 °C. The single-factor analysis of variance demonstrated a statistically significant relationship between the yield point obtained from both instruments with a correlation coefficient of 0.846 (*p* < 0.05). Then, in contrast to the TA instrument, which is unable to do so, the SSE-P can determine the yield point of the mass under study. Additionally, as there is a significant correlation between the results from SSE-P and those obtained from the rheometer, the yield point from SSE-P can be relied upon to pressurize the syringe before printing, thereby reducing the number of units that do not meet specifications.

These findings imply that most of the necessary information to manage the printing process may be obtained using the same printing platform, negating the need for additional tools like a rheometer. However, a rheometer is still irreplaceable for specific tests such us creep recovery or for fluid-type identification.

### 3.2. Printability and Fidelity Test

**Figure 5:**
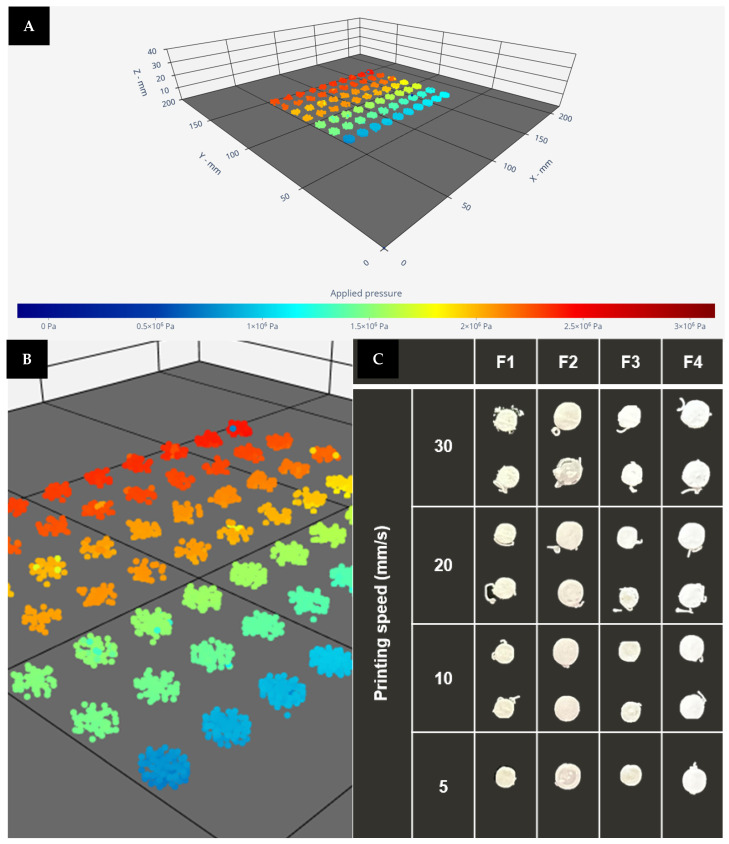
PAT applied to the 3DP process of the print cartridge. (**A**,**B**) Three-dimensional scatter for F1 and 5–30 mm/s. (**C**) Comparison of printlet for different formulations and printing speed.

Figure 5A shows the 3D scatter plot with the recorded pressure values located in the spatial coordinates for F1, and 6B shows the comparison of printlets depending on formulation and printing speed.

**Figure 6:**
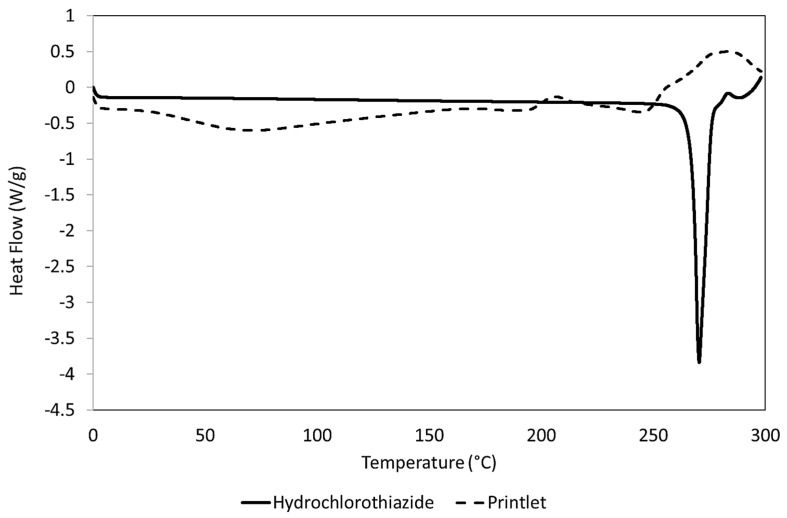
DSC thermal transition curves of pure hydrochlorothiazide and printlet.

In Figure 6 a DSC thermal transition curve of pure hydrochlorothiazide and a printlet is shown. Pure hydrochlorothiazide showed a melting endotherm peak at 270 °C which disappears due to its inclusion in the wet mass and, later, as part of the printlet. This is in line with what has already been published by other authors [12].

**Figure 7:**
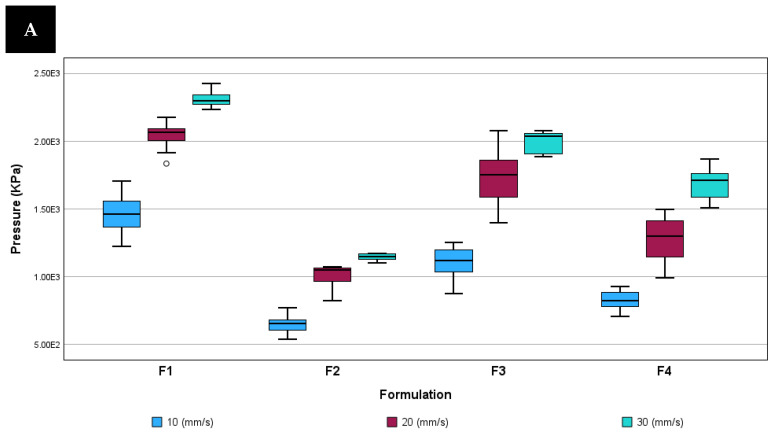

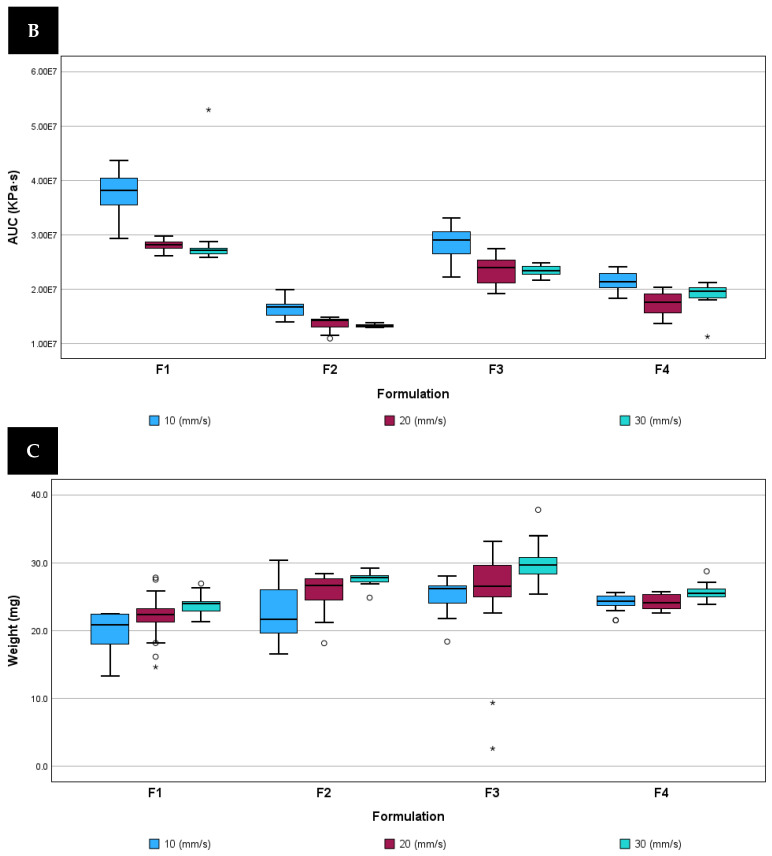

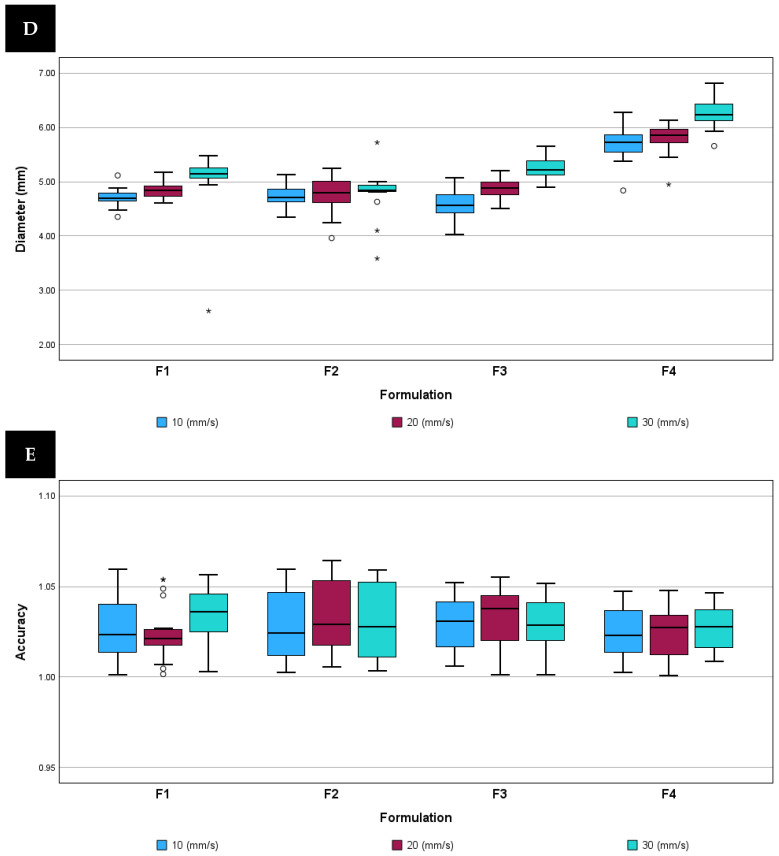
Box plot with outliers for each parameter, formulation, and printing speed studied. (**A**) Pressure; (**B**) area under the curve (AUC) (**C**) weight; (**D**) diameter; (**E**) accuracy. ○: Represents cases or rows with values greater than the height of the boxes multiplied by 1.5. *: Represents cases or rows with values greater than the height of the boxes multiplied by three.

Results for the printability test are shown in Figure 7 showing the most relevant parameters such as mean printing pressure during the printing process, total area under the curve per printlet obtained from force/time plot and the average weight of the dosage units for each printing speed and formulation.

As can be seen, there is a clear increase in printing pressure when printing speed rises, as more force is required to displace 1 mm of mass per second. Due to the same reason, a decrease in AUC was detected when printing speed was increased. In the case of tablet weight, a slight increase was detected during the rise in printing speed. This is related to the higher pressure applied and the energy remaining, which may increase the deposited mass. From the three printing speeds tested, 30 mm/s showed a lower standard deviation in mass weight for all formulations. This might be related to the fact that printlets elaborated at this speed were the last to be elaborated, and steady flow might be reached.

The analysis of data was performed using an analysis of variance and regression to demonstrate the relationship between two factors (formulation and printing speed) and the parameters previously mentioned (printing pressure, AUC, printlet weight, printlet diameter, and accuracy).

There was a statistically significant relationship between the increase in printing speed, and the changes in pressure, AUC, weight, and diameter of the resultant dosage units (*p* < 0.05). An HSD Tukey test was performed to confirm that the means for each variable from each group (10, 20, and 30 mm/s) were statistically different from each other by a pairwise comparison. In all cases, means were statistically different except for weight, where means for 10 and 20 mm/s showed little difference. In the case of formulations, they are considered statistically different in terms of pressure, AUC, and weight (except for F2 and F4). In the case of diameter, only values from F4 were considered different in comparison with the rest.

Printing speed is one of the printing parameters that has to be taken into account during printlet development as it has a great impact on the printing process, especially printing pressure and AUC. Those printing parameters could be used as part of a PAT strategy in order to ensure that production is between established limits and therefore under control.

The printing speed also influences CQAs such as tablet weight, which has a direct relation with API content. In this study, the difference between average weight of the dosage units from the same formulations printed at different speeds is less than 5 mg (at a maximum in F2), but it would be a relevant parameter to be evaluated in cases where the amount of API per dosage unit is low, for example, 1 mg, as in the case of the administration of Budesonide in paediatrics [36].

However, all of the issues previously mentioned can only be considered if the printing platform is instrumentalized with an SSE-P sensor, which allows the monitoring of relevant parameters such as printing pressure and enables the comparison with CQAs.

## 4. Conclusions

For the first time, a study has been carried out regarding the implications of the instrumentation of a 3DP platform by incorporating an SSE-P. This allowed the evaluation of the extrusion dynamics of different formulations in situ. It is far more reliable than other techniques in terms of mimicking printing process, as extrudability factors such as temperature or plunger speed can be modified. In addition, the results obtained were similar to traditional TA. However, SSE-P cannot fully replace the rheological characterization of the semi-solid mass as the identification of a Newtonian or non-Newtonian fluid is fundamental for printlet development in determining the buildability of the printing cartridge.

Moreover, the incorporation of this sensor, in combination with in-line and off-line quality control, made possible to evaluate changes in printing pressure when different formulations (but with similar compositions) and printing speeds are used. This has allowed the conclusion that there is a relationship between changes in critical printing parameters such as printing speed or formulations, and variations in printing pressure and printlets CQAs such as AUC, weight, and diameter.

Furthermore, the incorporation of a pressure sensor made it possible, as part of a PAT strategy, to monitor and control critical printing parameters such as temperature and printing speed and to establish relationship between them and CQAs. In addition, this would be of great benefit and may lead the way to the future incorporation of 3DP in point-of-care settings as the quality of printed dosage forms will be ensured.

## Figures and Tables

**Table 1:** Percentage composition of the starting semi-solid formulations (*w*/*w*).

	F1	F2	F3	F4
Hydrochlorothiazide	15.0	15.0	15.0	15.0
Lactose monohydrate	6.8	9.0	9.0	6.8
Polyvinyl pyrrolidone 30 K	3.0	3.0	3.0	3.0
Croscarmellose sodium	11.3	9.0	9.0	11.3
Purified water	62.8	64.0	62.9	63.9
Banana flavouring essence	1.1	0.0	1.1	0.0

**Table 2:** Compression/shrinkage cycle used to study material properties at different printing speeds: 10, 20, and 30 mm/s. * Amount of displacement (in mm) which the probe runs throughout the analysis during compression and shrinkage cycle.

Target Printing Speed (mm/s)		10	20	30
Step 1	Plunger speed (mm/s)	0.010	0.020	0.030
	Distance * (mm)	5	5	5
	Time (s)	500	250	166
Step 2	Plunger speed (mm/s)	Hold time		
	Distance * (mm)	Hold time		
	Time (s)	60	60	60
Step 3	Plunger speed (mm/s)	−0.100	−0.200	−0.300
	Distance * (mm)	5	5	5
	Time (s)	50	25	16
Step 4	Plunger speed (mm/s)	Hold time		
	Distance * (mm)	Hold time		
	Time (s)	60	60	60

**Table 3:** Yield point values obtained for each formulation at 40 °C.

Yield Point		
Formulation	Rheometer (Pa)	SSE-P (KPa)
F1	607	366
F2	1064	305
F3	198	417
F4	1064	363

**Table 4:** Influence of printing speed when TA is used at 25 °C.

Formulation	Printing Speed (mm/s)	Maximum Pressure (kPa)	Pressure Flow Cessation KPa)	Young Module (1/mm)	Shear Stress Steady Flow (KPa)	Apparent Viscosity (mPa·s)	Dynamic Viscosity (mPa·s)
F1	10	1094.5	900.3	204.1	1759.9	12.8	35.6
	20	1435.9	1022.8	303.1	2370.6	7.9	22.9
	30	1712.3	1141.0	405.9	2878.5	6.9	11.9
F2	10	511.9	340.9	222.5	901.4	6.3	17.5
	20	745.7	385.2	372.9	1237.2	4.3	12.0
	30	1033.6	575.2	328.6	1719.6	4,0	11.1
F3	10	678.6	425.0	349.7	1034.7	7.2	20.1
	20	814.3	505.3	292.9	1424.8	5.0	13.8
	30	1103.9	640.1	286.9	1698.8	3.9	11.0
F4	10	1056.8	757.7	401.7	1835.5	12.8	35.6
	20	1445.3	832.7	578.2	2478.5	8.6	24.0
	30	1525.2	864.1	462.6	2628.5	6.1	17.0

**Table 5:** Influence of printing speed when SSE-P is used at 25 °C.

SSE-P									
Formulation	Printing Speed (mm/s)	Yield Point (Kpa)	Yield Point Time (s)	Maximum Pressure (kPa)	Pressure Flow Cessation KPa)	Young Module (1/mm)	Shear Stress Steady Flow (KPa)	Apparent Viscosity (mPa·s)	Dynamic Viscosity (mPa·s)
F1	10	471.5	120.0	1402.3	1020.6	240.4	1993.7	13.9	38.6
	20	451.5	64.5	1611.2	1160.3	133.2	2248.9	7.8	21.8
	30	478.1	40.3	1979.9	1126.2	301.9	2767.7	6.4	18.0
F2	10	450.1	124.4	964.8	631.7	304.9	1473.6	10.2	28.6
	20	413.2	40.7	1261.2	744.6	711.4	1831.0	6.4	17.8
	30	438.8	35.0	1416.7	748.4	763.1	2243.5	5.2	14.5
F3	10	408.7	162.8	817.9	569.8	166.1	1114.0	7.7	21.6
	20	358.3	37.9	1170.6	567.5	178.4	1418.4	4.9	13.7
	30	334.8	23.3	1286.5	632.9	682.3	2293.9	5.3	5.5
F4	10	386.4	177.8	1082.3	797.8	170.0	1971.5	13.7	38.2
	20	415.0	77.3	2524.7	1647.5	214.6	2722.3	9.5	26.4
	30	405.9	44.5	2083.5	1158.9	509.4	3663.3	8.5	23.7

## Data Availability

The data can be shared up on request.

## Abbreviations

3DP	Three-dimensional printing
DPE	Direct powder extrusion
MED	Melt extrusion deposition
FDM	Fused deposition modelling
SSE	Semi-solid extrusion
API	Active pharmaceutical ingredient
CQA	Critical quality attribute
TA	Texture analyser
SSE-P	Semi-solid motor-driven printhead
PAT	Process analytical technology
HCTZ	Hydrochlorothiazide
PVP	Polyvinylpyrrolidone
PDS	Plunger displacement speed
TPS	Target print speed
LH	Layer height
D	Syringe inner diameter
γ	Shear rate
R	Nozzle radius
Q	Volumetric flow rate
F	Force
µ	Dynamic viscosity
A	Syringe area
L	Tip length
D	Tip diameter
AUC	Area under the curve
